# Photocapacitive CdS/WO_x_ nanostructures for solar energy storage

**DOI:** 10.1038/s41598-019-48069-5

**Published:** 2019-08-09

**Authors:** Daniel R. Jones, Robert Phillips, William J. F. Gannon, Bertrand Rome, Michael E. A. Warwick, Charles W. Dunnill

**Affiliations:** Energy Safety Research Institute (ESRI), Swansea University Bay Campus, Swansea, SA1 8EN UK

**Keywords:** Energy harvesting, Energy storage

## Abstract

Through a facile solvothermal procedure, a CdS/WO_x_ nanocomposite has been synthesised which exhibits photocapacitive behaviour under white light illumination at a radiant flux density of 99.3 mW cm^−2^. Photoelectrochemical experiments were undertaken to examine the self-charging properties of the material and to develop an understanding of the underlying electronic band structure responsible for the phenomenon. By employing XPS, UPS and UV-Vis diffuse reflectance spectroscopy for further characterisation, the ability of the composite to generate current following the removal of incident light was related to the trapping of photoexcited electrons by the WO_x_ component. The presence of WO_x_ yielded an order of magnitude increase in the transient photocurrent response relative to CdS alone, an effect attributed to the suppression of electron-hole recombination in CdS due to hole transfer across the CdS/WO_x_ interface. Moreover, current discharge from the material persisted for more than twenty minutes after final illumination, an order of magnitude improvement over many existing binary composites. As a seminal investigation into the photocapacitive characteristics of CdS/WO_x_ composites, the work offers insight into how the constituent materials might be utilised as part of a future self-charging solar device.

## Introduction

As a consequence of the global shift towards renewable sources of energy, the development of energy storage technologies has grown increasingly important. Indeed, if the world is to substitute traditional fossil fuels for variable and unpredictable alternatives such as solar or wind power, energy from these sources must be stored during periods of surplus supply for times of excess demand. To this end, consumers are beginning to explore the benefits of household battery systems as a storage medium for solar energy^1–3^, and there are also multiple larger-scale energy storage projects currently planned or underway across the globe^4^. An alternative solution is the use of electrolysers to store surplus energy in the form of hydrogen^5–7^, a commodity which may be used to generate electricity directly through use of fuel cells^8,9^ or otherwise combusted in the manner of a conventional fuel gas^10^.

Despite the practicability of existing storage technologies, their widespread implementation is inhibited by factors such as the financial cost of necessary infrastructure^11–14^ and their unsuitability in size-limited applications^15,16^. The prospect of *in situ* energy storage is therefore an attractive one: by simultaneously generating electrical power and storing surplus energy within a single device, the need for an external storage medium is bypassed. The potential of such an approach has been most notably showcased by photocapacitive “self-charging” solar cells^15–29^, which serve as a source of electrical power under solar illumination whilst simultaneously retaining a proportion of the incident energy in the form of a stored electrical charge. The earliest example of a such a device incorporated titanium dioxide as a UV-absorbing component and a supercapacitor comprised of layers of activated carbon with a porous separator, all integrated into a single monolithic device^24^. The success of a self-charging solar cell depends not only on the physical and chemical properties of its photoactive and supercapacitive components, but also the intimacy of the electronic junction between them.

In the present investigation, the photocapacitive behaviour of a nanocomposite of cadmium sulphide, CdS, and tungsten(VI) sub-oxide, WO_x_, is explored under white light illumination at a power density of approximately one Sun. The constituents of this composite, which shall be henceforth referred to as CdS/WO_x_, were selected for their respective photoabsorbance and surface-reactivity characteristics; CdS, for instance, has a typical photonic band-gap of 2.4 eV^30–33^, and is consequently a proficient absorber of visible light. Moreover, the electron mobility in CdS is typically in the range 1–10 cm^2^ V s^−1^, similar to reported values for TiO_2_^34–38^. A primary drawback of CdS, however, is its susceptibility to photocorrosion, although the phenomenon may be suppressed through the efficient scavenging of photoinduced holes^39–44^. By incorporating the material into a composite of strategically-selected constituents, it is therefore possible to improve the stability of CdS whilst preserving its desirable properties.

As the second component of the composite, WO_x_ typically provides a high surface area due to the propensity of the material to form crystalline nanostructures^45^, and the intrinsic oxygen vacancies function as both reactive surface sites^46–49^ and electron donor states^50–52^, as well as allowing the material to operate as an effective electron sink^53–58^. By employing a solvothermal approach used previously by the present researchers^59^, WO_x_ nanostructures may be grown heterogeneously from the surface of pre-synthesised particles to promote an intimate junction between the constituent materials. Herein, the CdS/WO_x_ nanostructures so-formed are tested using a three-electrode photoelectrochemical setup to verify their applicability to photocapacitive applications, exploring the electrical behaviour during and after white light exposure and the stability of the response following prolonged illumination. The study aims not to demonstrate the function of a completed self-charging solar cell, but rather it serves to identify the physical interplay between CdS and WO_x_ and to elucidate how a workable self-charging CdS/WO_x_ device might be realised.

## Results

### Material characterization

**Figure 1:**
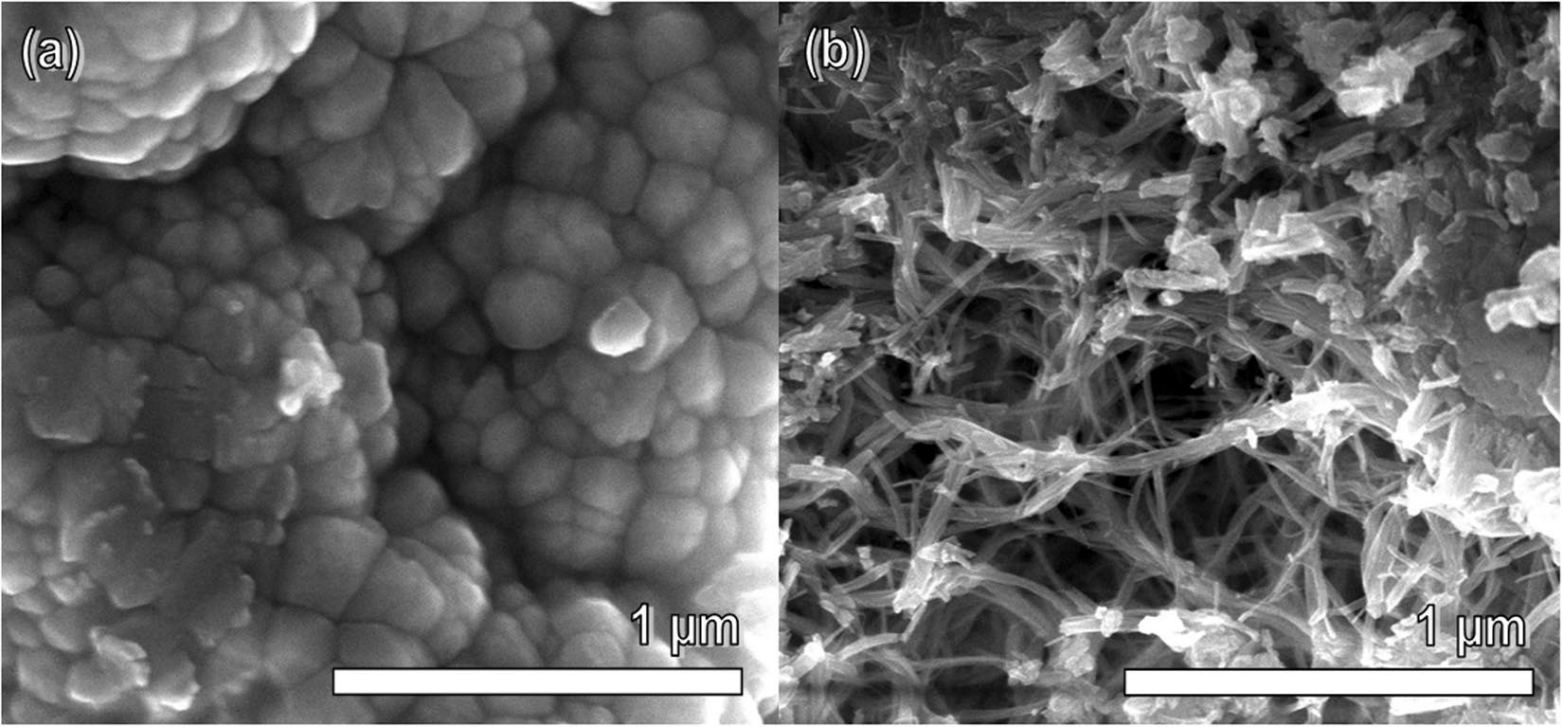
SEM images depicting the aggregated nanoparticles of CdS (**a**) and the WO_x_ nanowires present in CdS/WO_x_ (**b**); the WO_x_ nanowires resemble the corresponding WO_x_ nanostructures observed in a core-shell Ta_3_N_5_/WO_x_ composite from a previous report^59^, which is employed herein as a reference sample.

As shown by the SEM image in Fig. 1a, the solvothermal synthesis of CdS yielded nanoparticles with a typical diameter of less than 200 nm, although it was difficult to acquire precise size estimates due to particle aggregation. Subsequent heterogeneous growth of WO_x_ sub-oxides on the CdS surface yielded the morphology depicted in Fig. 1b; the WO_x_ nanowires shown in this SEM image are characteristic of those grown solvothermally in the formation of other composites^59–62^, most notably the Ta_3_N_5_/WO_x_ sample synthesised via a near-identical protocol in a previous report by the present group^59^. Making use of this similarity between the CdS/WO_x_ and Ta_3_N_5_/WO_x_ composites, the latter is employed as a reference sample in the present study to facilitate understanding of how photoelectrochemical measurements from CdS/WO_x_ are related to electronic interactions between the two components.

**Figure 2:**
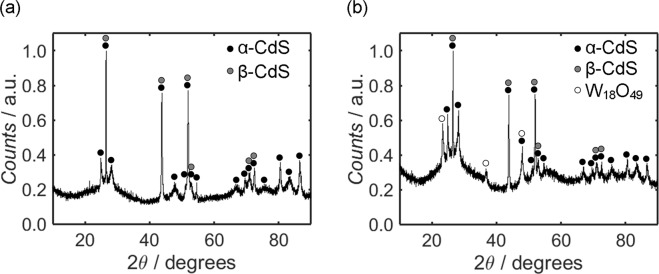
XRD diffractograms measured from CdS (**a**) and CdS/WO_x_ (**b**), with the peaks indexed to α-CdS (JCPDS number 41–1049), β-CdS (JCPDS number 10–0454) and W_18_O_49_ (JCPDS number 71–2450) indicated by black, grey and white circles, respectively.

A key consideration for an electrode material is its crystallinity, which may be characterised through use of XRD. Annotated on the diffractograms of CdS (Fig. 2a) and CdS/WO_x_ (Fig. 2b) are the peaks corresponding to the hexagonal wurtzite α-CdS phase, otherwise known as greenockite (with peaks identified by black circles)^63–71^, cubic sphalerite β-CdS, or hawleyite (identified by grey circles)^66–71^, and monoclinic W_18_O_49_ (marked by white circles)^72–74^, as indexed by JCPDS reference cards 41–1049, 10‐0454 and 71–2450, respectively. Due to overlaps between the diffractogram peaks of α-CdS and β-CdS, it is difficult to discern the relative composition of the two phases; the high relative intensity of the peak at 26.5°, however, implies that both phases were present in the samples. The diffuse background in each diffractogram is indicative of disorder within the material associated with lattice defects; this feature is most clearly apparent in the case of CdS/WO_x_, and the diffuse scattering is possibly attributable to oxygen vacancies within the WO_x_ component^75^.

**Figure 3:**
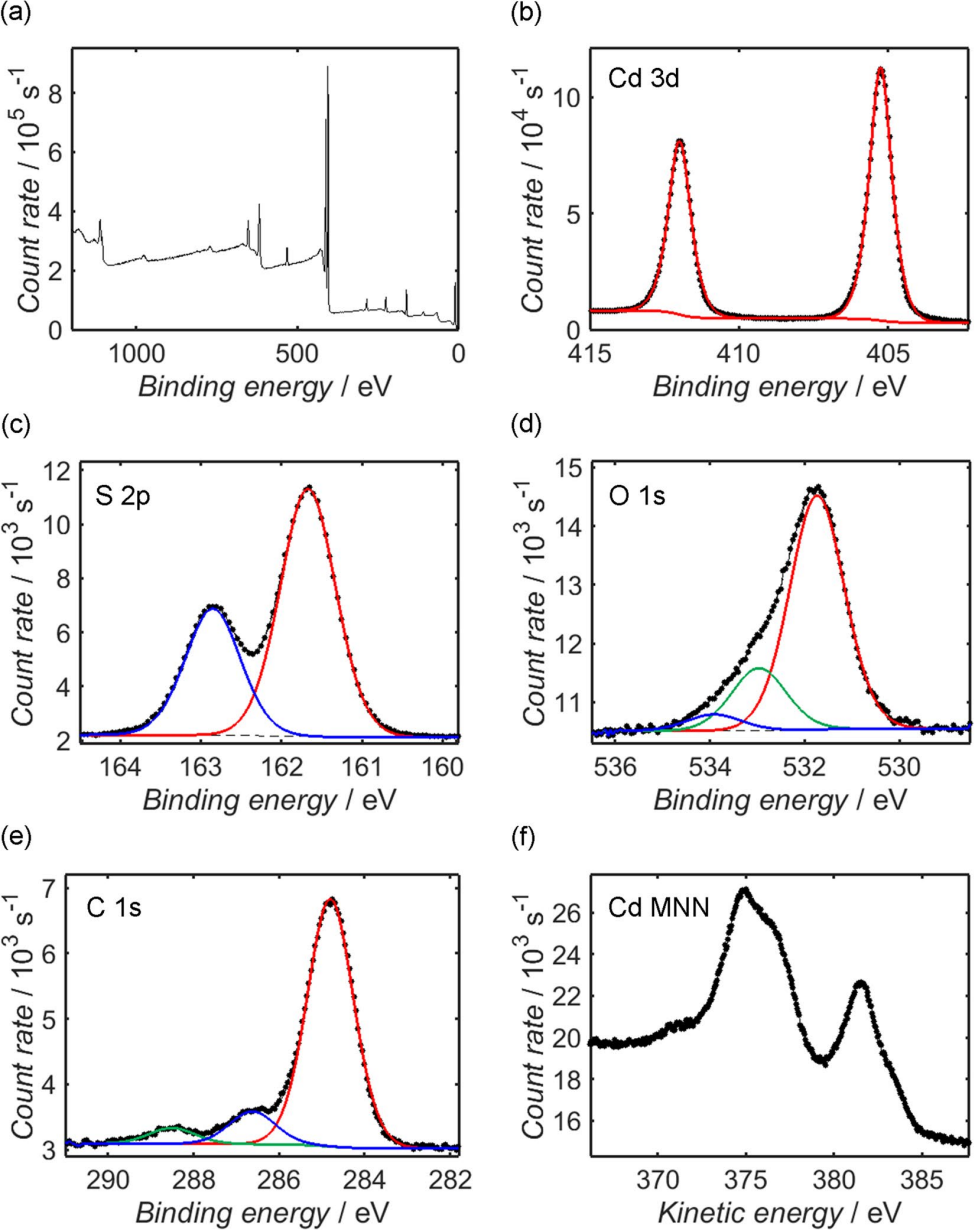
XPS measurements from the CdS sample over the range 0–1200 eV (**a**), in addition to higher-resolution measurements of the Cd 3d (**b**), S 2p (**c**), O 1 s (**d**) and C 1 s (**e**) photoelectron peaks and the Cd MNN Auger spectrum (**f**). The secondary electron background of each photoelectron peak is modelled by a Shirley-type function, plotted in each case as a dashed black line, while the peaks themselves are deconvoluted into Gaussian-Lorentzian components, depicted as solid coloured lines, with components within the same peak doublet assigned the same colour.

**Figure 4:**
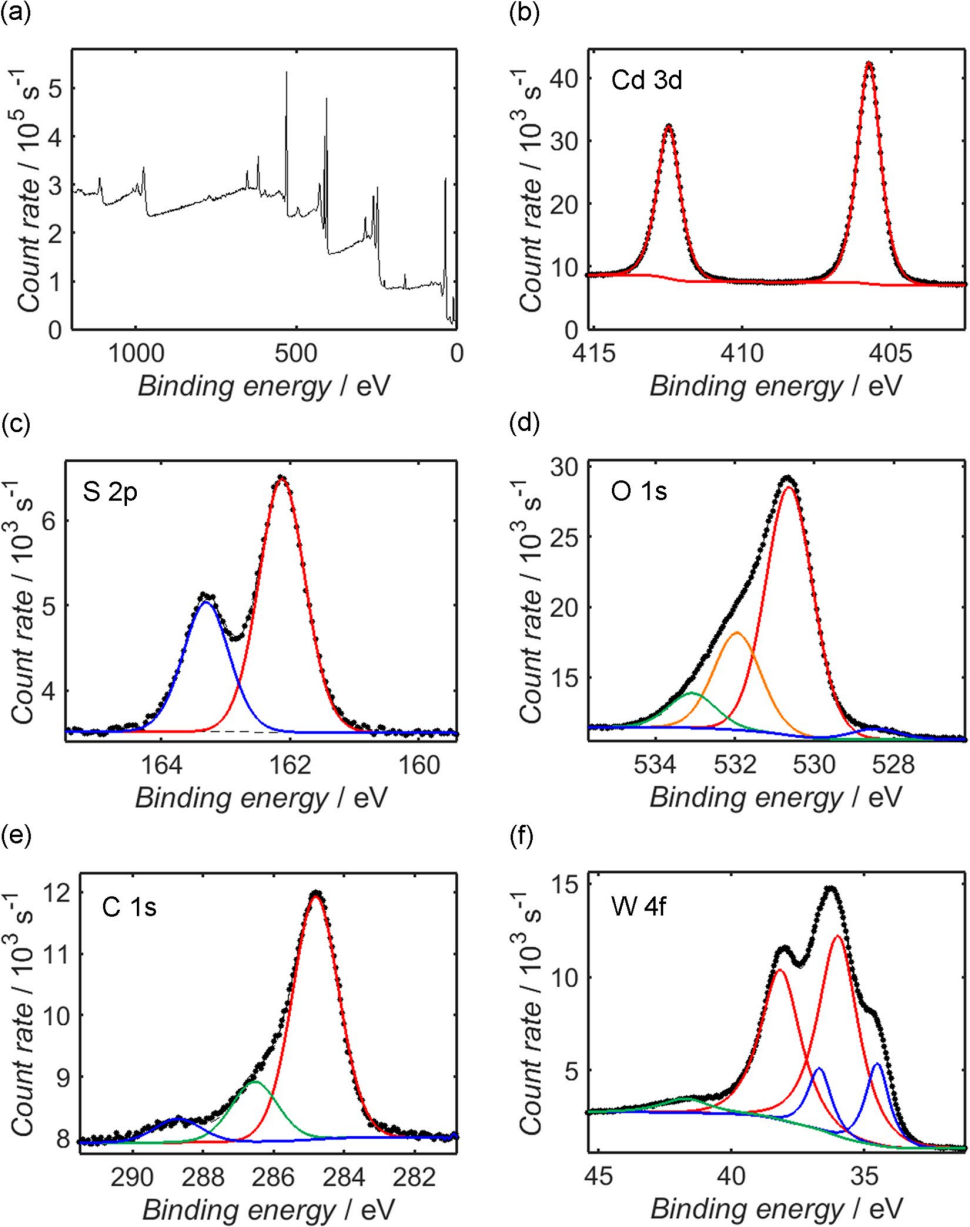
XPS measurements from the CdS/WO_x_ composite over the range 0–1200 eV (**a**), alongside higher-resolution measurements of the Cd 3d (**b**), S 2p (**c**), O 1 s (**d**), C 1 s (**e**) and W 4f (**f**) photoelectron peaks. Shirley-type fits to the secondary electron background of each photoelectron peak are plotted as dashed black lines, while the fitted Gaussian-Lorentzian peak components are depicted as solid coloured lines; components within the same peak doublet are assigned the same colour. It should be noted that due to differential charging between the CdS and WO_x_ components, the carbon-correction applied to the peaks of CdS is likely unsuitable; for instance, the binding energy positions of the S 2p and Cd 3d peaks are approximately 0.5 eV higher than the corresponding values in Fig. 3.

The surface chemical composition of each sample was investigated using XPS. The survey spectrum of CdS (Fig. 3a) is accompanied by higher-resolution scans of the Cd 3d (**b**), S 2p (**c**), O 1 s (**d**) and C 1 s (**e**) photoelectron peaks, as well as the Cd MNN Auger peak (**f**), while the survey spectrum of CdS/WO_x_ (Fig. 4a) is displayed alongside scans of the Cd 3d (**b**), S 2p (**c**), O 1 s (**d**), C 1 s (**e**) and W 4f (**f**) photoelectron peaks. The measured data in each case is represented by circular black markers connected by straight lines, with dashed black lines used for both the Shirley background function and the fitted envelope obtained from the sum of the coloured Gaussian-Lorentzian fitting components; for ease of interpretation, components corresponding to the same peak doublet are plotted in the same colour, while alternative colours are used to represent different chemical environments.

In the case of the CdS sample, it is clear that both the Cd 3d and S 2p peaks are well-fitted by a single pair of doublet components with spin-orbit separation values of 6.74 eV^76,77^ and 1.18 eV^78,79^, respectively, and the form of the Cd MNN Auger signal is characteristic of CdS^80,81^. The purity of the CdS surface is belied, however, by the relative areas of the Cd 3d and S 2p peaks: the measured Cd/S atomic ratio of 1.42 is significantly higher than the expected value of unity, indicating the presence of other Cd-containing compounds. Indeed, the form of the O 1 s peak provides further evidence of these surface species: the component centred at 531.7 eV is consistent with compounds such as Cd(OH)_2_ or CdCO_3_^66,82–87^, while the adjacent contribution at 533.0 eV has previously been attributed to OH^−^ ions intercalated within the CdS lattice^86^. The final component, centred at a binding energy of 533.9 eV, is typically associated with adsorbed water^66,85,86^. It should be noted that adventitious organic species likely also contribute to the O 1 s spectrum, particularly the component at 533.0 eV^88,89^, and are also responsible for the sizeable C 1 s peak; this signal may be deconvoluted into a principal component at 284.8 eV, which is associated with C atoms contained within an aliphatic hydrocarbon chain, and secondary components at 286.6 eV and 288.5 eV, corresponding to C-O and O-C=O groups, respectively^89^. Ignoring the adventitious organic layer, XPS deconvolution yields a tentative estimate of surface composition: assuming that the Gaussian-Lorentzian components have been accurately designated, CdS comprised approximately 70.3% of the surface layer, with other compounds such as Cd(OH)_2_ accounting for the remainder. Nevertheless, EDX measurements from the CdS sample, which are displayed in Fig. S1a of the Supplementary Information alongside the estimated elemental composition in Supplementary Table S1, show that the O/C atomic ratio is consistent with the corresponding value from the underlying carbon tab, while the Cd/S atomic ratio has an estimated value of 1.04; these results suggest that whilst Cd-containing contaminants were present at the surface of each nanoparticle, the bulk consisted predominantly of CdS.

A similar analysis may be applied to CdS/WO_x_. The appearance of significant Cd 3d and S 2p peaks in Fig. 4 suggests that CdS was present at the composite surface: comparison of the W 4f and Cd 3d signals reveals a Cd/W atomic ratio of 0.58, implying that a high proportion of the CdS surface was exposed to the aqueous electrolyte during photoelectrochemical testing. Since the Cd/S atomic ratio from the CdS/WO_x_ spectra has a value of 1.40, one may further infer that the CdS surface remained unaffected by the formation of WO_x_; indeed, EDX measurements from the CdS/WO_x_ sample, shown in Fig. S1b of the Supplementary Information, provide support for this suggestion, as the measured elemental composition, detailed in Supplementary Table S1, reveals a bulk Cd/S atomic ratio estimate of 1.06, which is consistent with the corresponding value for CdS. One should note from the EDX spectrum that there is a greater abundance of O within the CdS/WO_x_ sample than anticipated from WO_x_ alone, with the surplus accounting for over half of the overall O content; since it is not possible to reconcile this result with the underlying carbon tab, which was shown to contain O at a concentration of approximately 7 at%, it is likely that preparation of the composite resulted in residual organic compounds that were not removed during subsequent centrifugation steps.

Despite the likelihood that Cd(OH)_2_ and other possible Cd-containing species contributed to the O 1 s peak of the CdS/WO_x_ XPS spectrum, it should be recognised that the absolute area of the peak is 5.3 times greater than the O 1 s signal of CdS, while the Cd 3d peak of Cd/WO_x_ is smaller than the corresponding peak of CdS by a factor of 3.0. One may therefore deduce that within the CdS/WO_x_ sample, O 1 s contributions from the CdS surface accounted for roughly one-sixteenth of the total, and hence it is reasonable to neglect them during deconvolution of the O 1 s spectrum. The O 1 s component centred at 530.6 eV may be attributed to O atoms within the WO_x_ lattice, while the component at 531.9 eV corresponds to O atoms at the surface of the WO_x_ lattice and the highest energy contribution, at 533.1 eV, is again typically associated with surface species such as adsorbed water^90–96^. The small component centred at 528.5 eV cannot be reconciled with WO_x_, but it is consistent with CdO^66,83–86^; no such component was observed in the O 1 s signal of CdS, suggesting that Cd(OH)_2_ or other Cd-containing compounds at the CdS surface were converted to CdO during formation of the composite.

Due to partial reduction of the W^6+^ ions as WO_x_ was formed, lower oxidation states of W must be considered during deconvolution of the W 4f signal. Assigning a doublet to each chemical environment, and constraining the spin-orbit separation to 2.17 eV^97^ in each case, the signal is well-represented by two pairs of components, with a small additional singlet at higher binding energy identified as the W 5p_3/2_ peak^95,96^. The primary contributor to the W 4f spectrum is the W^6+^ doublet, within which the 4f_7/2_ state is situated at a binding energy of 36.0 eV, while the remainder of the signal is accounted for by the W^5+^ doublet, which has its 4f_7/2_ state centred at 34.5 eV^90–96^; these component positions are in close agreement with the corresponding values of the Ta_3_N_5_/WO_x_ composite characterised previously^59^, which is employed herein as a reference material. By comparing the combined areas of the W^6+^ and W^5+^ doublets to the WO_x_ components of the O 1 s signal, one obtains a W/O atomic ratio estimate of 2.75, in close agreement with the value of 2.72 that is consistent with the formula W_18_O_49_.

**Figure 5:**
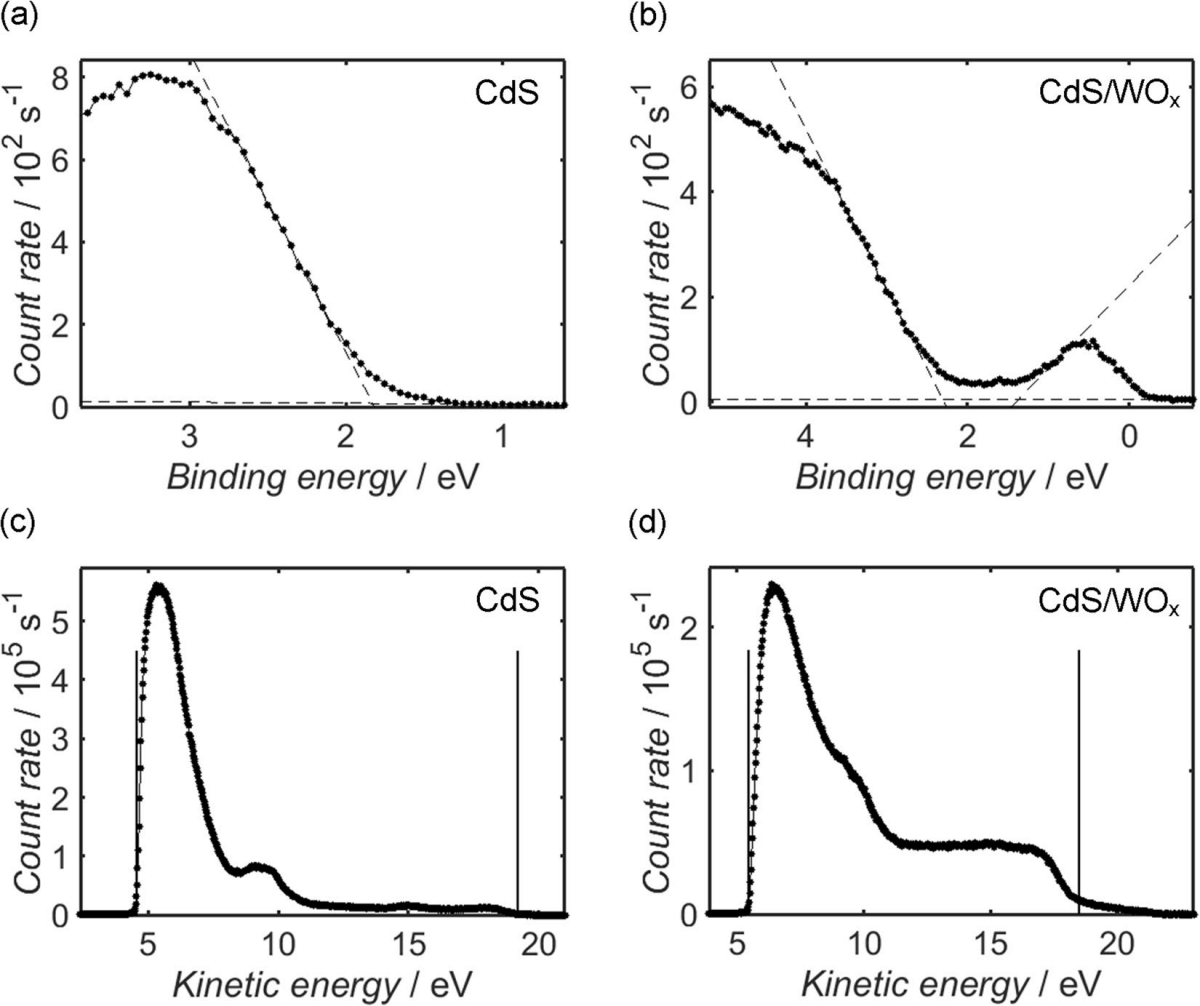
XPS measurements from CdS (**a**) and CdS/WO_x_ (**b**) close to the Fermi level, which is calibrated to a binding energy of 0 eV, in addition to UPS measurements from the same CdS (**c**) and CdS/WO_x_ (**d**) samples acquired using He I radiation (photon energy 21.22 eV). The dashed lines in the XPS scans represent the linear fits used to estimate the binding energy of the valence band maximum, or, in the case of CdS/WO_x_, the conduction band minimum, which are estimated from the intersection of the relevant line with a linear fit of the baseline data. To determine the position of the valence band maximum relative to the vacuum level (otherwise known as the ionisation potential), the valence band edge and secondary electron onset in each UPS spectrum are estimated according to the protocol illustrated in Fig. S2 of the Supplementary Information; the kinetic energies of these thresholds are identified in each spectrum by vertical black lines, and the ionisation potential is estimated by subtracting the difference between them from the incident photon energy.

It is evident from Fig. 4b,c that the positions of the Cd 3d and S 2p peaks of CdS/WO_x_ are offset by approximately 0.5 eV with respect to their CdS counterparts in Fig. 3b,c. One should note that the spectra of both samples have been corrected by referencing the C 1 s peak to its accepted value of 284.8 eV; it is probable, therefore, that the systematic disparity in the Cd 3d and S 2p peak positions is indicative of differential charging between the CdS and WO_x_ components of the composite, which in turn suggests that there was a significant potential barrier between the two phases at the sample surface. Indeed, the same potential offset is visible in XPS valence band measurements depicted for CdS and CdS/WO_x_ in Fig. 5a,b, respectively: through linear extrapolation of the valence band edge to an analogous fit of the baseline data^98–100^, the valence band maximum of the CdS sample, *E*_VB_(CdS), is estimated as 1.8 eV relative to the Fermi level of the instrument (which is calibrated to 0 eV), compared to 2.3 eV in the case of CdS/WO_x_. It has been noted by other researchers that within a composite of photoactive and charge-trapping components, such a Schottky barrier at the inter-component interface may assist with the effective trapping of photoexcited electrons and thereby promote electron-hole separation^101^, in turn increasing the average lifetime of these photogenerated charges.

The appearance of electronic states close to the Fermi level in Fig. 5b is a common occurrence in WO_x_ sub-oxides^102–104^, and the feature is indicative of populated conduction band states and associated degenerate semiconductor behaviour. By applying a linear fit to the edge of the sub-band feature, the position of the conduction band minimum of WO_x_, *E*_CB_(WO_x_), may be estimated relative to the Fermi level of the instrument; assuming that the estimate of *E*_VB_(CdS) from Fig. 5b is offset by 0.5 eV due to differential charging with respect to the WO_x_ component, the measurements are consistent with a difference of approximately 0.4 eV between *E*_VB_(CdS) and *E*_CB_(WO_x_) if electronic equilibrium were to exist between the components. From these relative band edge positions one may infer that electron-hole recombination between the conduction band of WO_x_ and valence band of CdS was thermodynamically feasible; this process of interfacial electron-hole recombination acts to suppress alternative recombination of photoexcited electrons within the photoactive phase, thereby increasing the mean lifetime of such electrons and promoting photocurrent^105,106^. It should be recognised that since the potential offset between CdS and WO_x_ resulted in an increased difference between *E*_VB_(CdS) and *E*_CB_(WO_x_) relative to the case of electronic equilibrium between the components, the effect conceivably facilitated the process of electron-hole recombination across the inter-component interface.

The valence band maximum at the surface of each sample was further investigated using UPS; spectra from the argon-cleaned CdS and CdS/WO_x_ samples are shown in Fig. 5c,d, respectively. By employing a protocol described elsewhere^59^, as detailed by Fig. S2 of the Supplementary Information and the accompanying discussion, *E*_VB_ may be determined in each case from the energy difference between the secondary electron onset, *E*_k,SEO_, and the cut-off at the valence band edge, *E*_k,VB_: subtraction of *E*_k,VB_-*E*_k,SEO_ from the photon energy, *hν*, yields an absolute value for *E*_VB_ relative to the vacuum level, *E*_vac_. One should note that whilst UPS is a highly surface-sensitive technique, the obtained *E*_k,VB_-*E*_K,SEO_ estimates are typically representative of bulk values in non-degenerate semiconductors because the edges of the conduction and valence bands are fixed with respect to *E*_vac_, provided that the Fermi level is not pinned by surface states^107–111^. Previous research suggests that CdS may be assumed to satisfy this condition^111–114^, whereas the aforementioned bulk degeneracy of WO_x_ is inconsistent with the standard Schottky-Mott model of band-bending; nevertheless, the occupation of conduction band states in degenerate systems precludes the formation of a space-charge region^109^, allowing surface band-bending to be discounted.

Based on the measurements in Fig. 5c, *E*_VB_(CdS) is estimated as 6.6 eV relative to vacuum, while Fig. 5d yields a corresponding estimate of 8.2 eV for the valence band maximum of the CdS/WO_x_ composite. Despite the appearance of signals from both CdS and WO_x_ in the XPS spectrum of CdS/WO_x_, it is instructive to note that the *E*_k,VB_ and *E*_k,SEO_ values from Fig. 5d are similar to corresponding estimates for Ta_3_N_5_/WO_x_ and WO_x_ obtained in a previous report;^59^ this similarity indicates that the *E*_VB_ estimate from Fig. 5d is in turn a close approximation of the valence band maximum in WO_x_, and the value shall therefore be henceforth denoted *E*_VB_(WO_x_).

**Figure 6:**
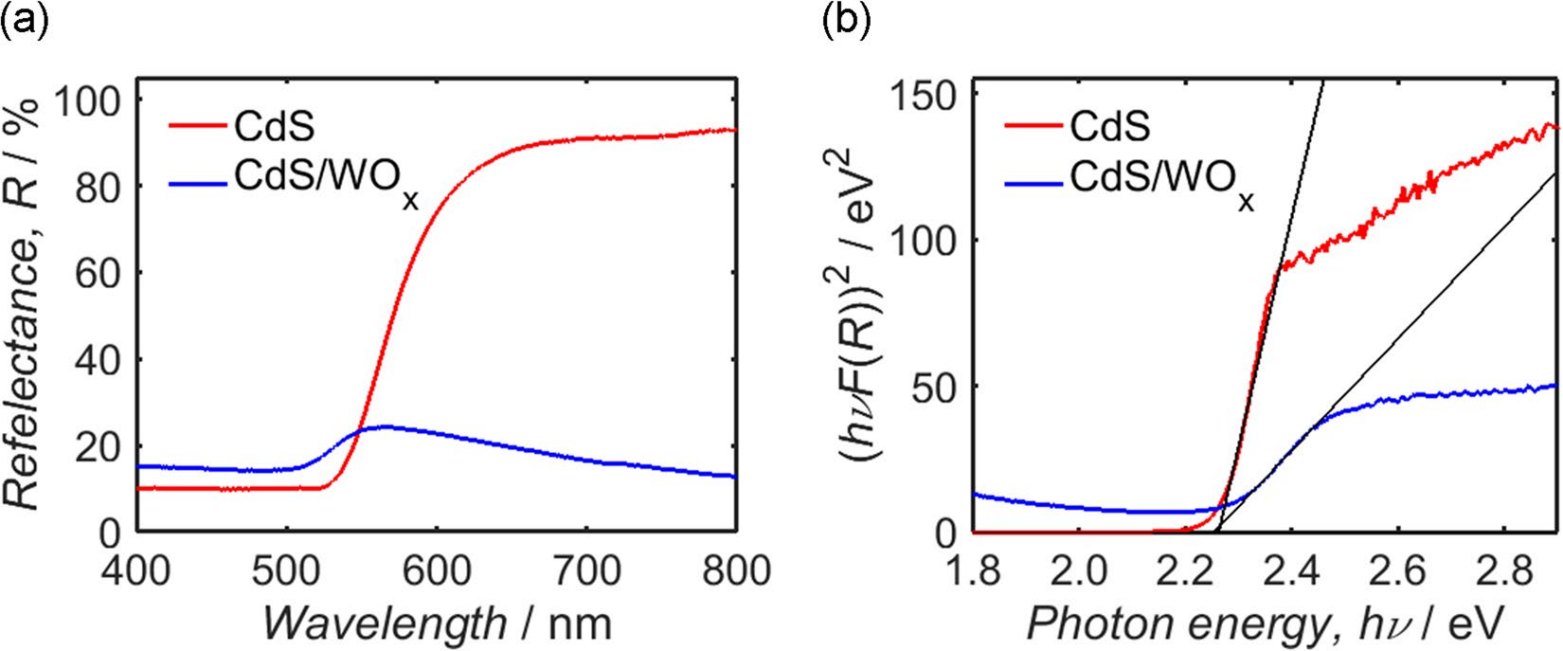
UV-Vis diffuse reflectance spectra (**a**) and the corresponding Tauc plots (**b**) of CdS and CdS/WO_x_. In each Tauc plot, the photonic band-gap is estimated as the x-intercept of a linear fit through the point of maximum gradient, depicted in each case as a solid black line.

To determine the photonic band-gap of each sample, UV-Vis diffuse reflectance measurements are next addressed. The diffuse reflectance, *R*, of each sample, is plotted as a function of incident wavelength in Fig. 6a; by calculating the Kubelka-Munk function^33,115^, *F*(*R*), as a function of photon energy, *hν*, Tauc plots have been constructed from these measurements, as shown in Fig. 6b. Within each Tauc plot, a Matlab program was used to construct a linear fit through the points of steepest gradient, the x-intercept of which provides an estimate of the photonic band-gap; the plots of CdS and CdS/WO_x_ yield comparable values of 2.26 eV and 2.25 eV, respectively, which are consistent with CdS band-gap estimates quoted elsewhere in the literature^55,66,71,105,116–118^. Recalling the value of *E*_VB_(CdS) from Fig. 5c, the conduction band minimum of CdS, *E*_CB_(CdS), is thus estimated as 4.3 eV relative to *E*_vac_. As *E*_CB_(WO_x_) cannot be estimated relative to *E*_vac_ from the present results, a difference of 1.6 eV between *E*_CB_(WO_x_) and *E*_VB_(WO_x_) is to be assumed in accordance with previous work^59^.

**Figure 7:**
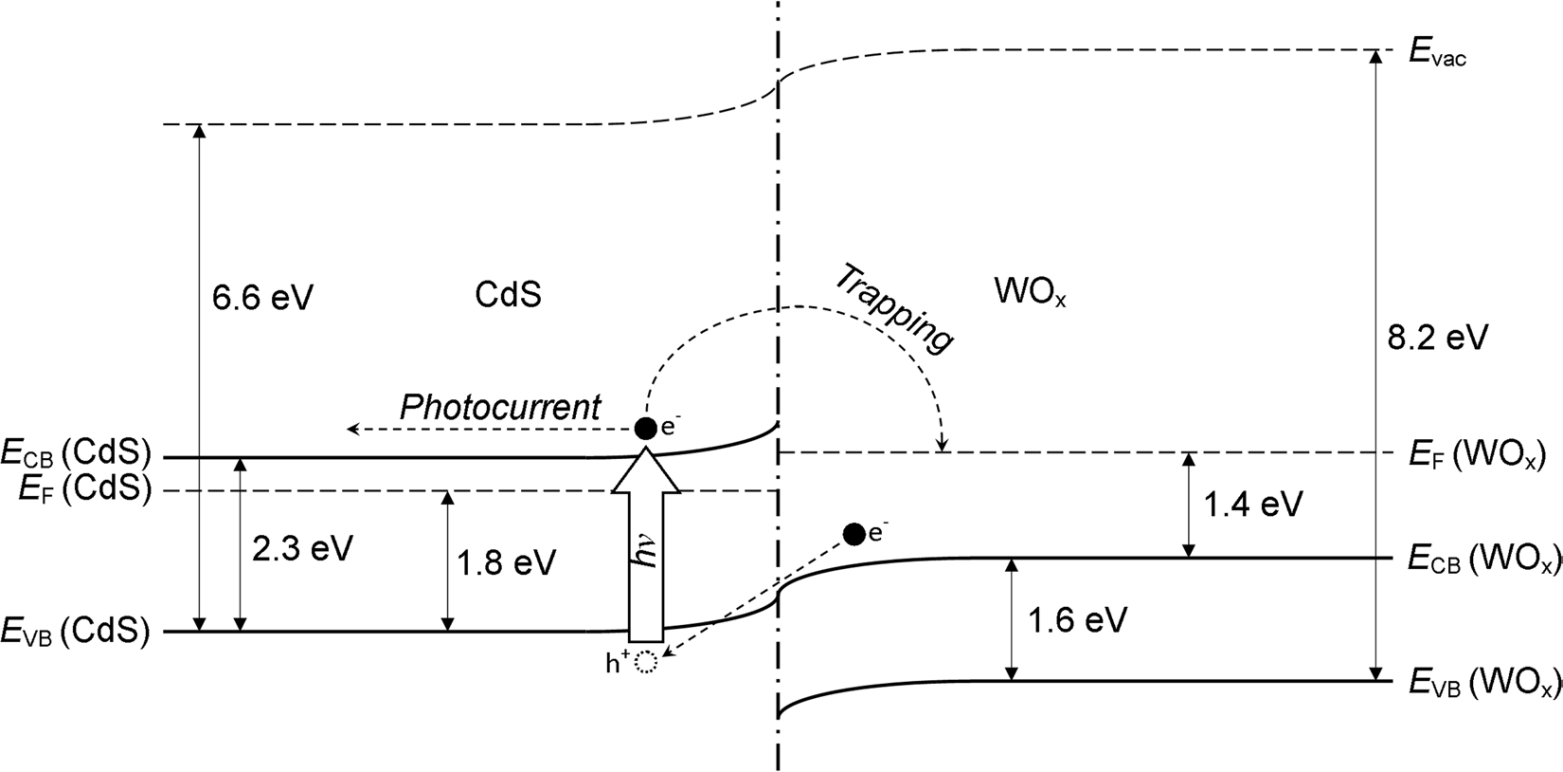
Proposed band structure of the CdS/WO_x_ composite, constructed using energy estimates from XPS, UPS and UV-Vis diffuse reflectance measurements. A potential offset of 0.5 eV has been assumed between the Fermi levels of CdS and WO_x_, denoted *E*_F_(CdS) and *E*_F_(WO_x_), respectively, in accordance with the XPS measurements in Figs 4, 5; it should be recognised that these values are not representative of the material in other electronic environments, but are nevertheless instructive for qualitative determination of the relative energy band edge positions. Based on the plotted estimates, an energy difference of approximately 1.4 eV is predicted to have existed between the conduction band minima of CdS, *E*_CB_(CdS), and WO_x_, *E*_CB_(WO_x_), which indicates that the inter-component transfer and subsequent trapping of photoexcited electrons was thermodynamically viable. The trapping of photoexcited electrons and their contribution to photocurrent was likely facilitated by the quenching of photoinduced holes by electrons from the valence band of WO_x_, as shown.

By combining the values of *E*_CB_(CdS) and *E*_CB_(WO_x_) with the aforementioned estimates obtained from Fig. 5, one may construct a rudimentary schematic of the band positions in CdS/WO_x_, as illustrated in Fig. 7. One should acknowledge that whilst the values of *E*_VB_ and *E*_CB_ relative to *E*_vac_ are generally applicable, the Fermi level positions of CdS and WO_x_, denoted *E*_F_(CdS) and *E*_F_(WO_x_), respectively, are dependent on the environment in which the composite is placed. Nevertheless, the *E*_F_ estimates derived from XPS may be used to provide insight into the relative positions of the energy band edges, and for this reason a difference of 0.5 V between *E*_F_(CdS) and *E*_F_(WO_x_) has been assumed in the diagram, in accordance with the measured potential offset between the two components in Figs 4, 5. As the plotted values of *E*_F_(CdS) and *E*_F_(WO_x_) are not representative of the composite in the presence of electrolyte, the case of electronic equilibrium between the constituents shall also be addressed.

According to the estimated band positions in Fig. 7, the conduction band edge of CdS was situated approximately 1.4 eV above the minimum of the WO_x_ conduction band, while this difference increases to 1.9 eV if one instead imposes the condition of electronic equilibrium between the components. In both scenarios, therefore, the transfer of photoexcited electrons from CdS to WO_x_ was thermodynamically viable, permitting the accumulation of photogenerated charge at trap sites in the WO_x_ phase. Also shown in Fig. 7 is the possible recombination of electrons from the conduction band of WO_x_ with photoinduced holes in the valence band of CdS, thereby suppressing their recombination with photoexcited electrons; following their promotion to the conduction band of CdS, these electrons were able to either contribute to photocurrent or migrate to WO_x_ and occupy trap sites present therein.

### Photoelectrochemical tests

**Figure 8:**
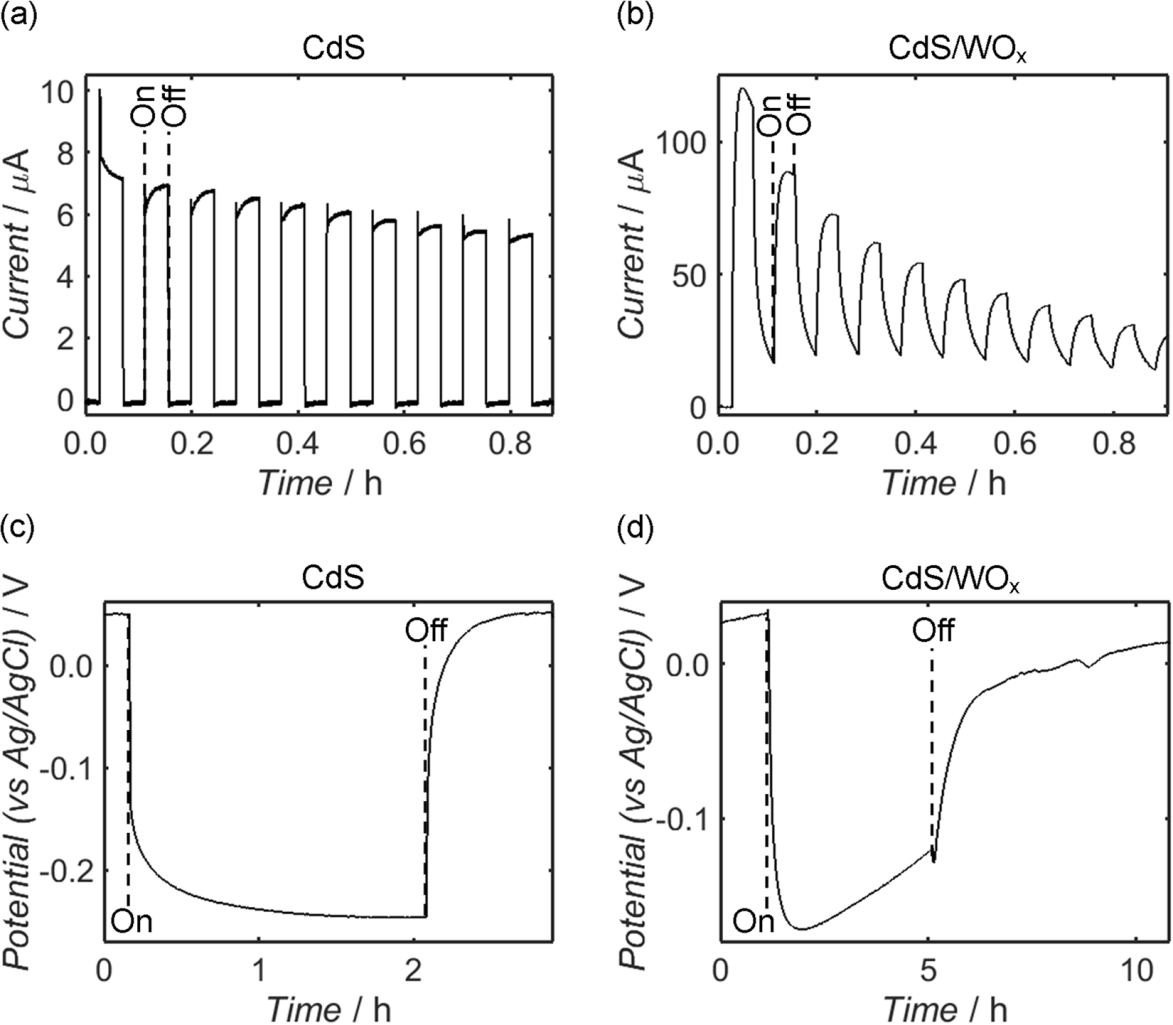
Photocurrent response measurements from CdS (**a**) and CdS/WO_x_ (**b**) on FTO-coated glass in a three-electrode configuration with a platinum mesh counter electrode and Ag/AgCl (3.0 M) reference, alongside open-circuit potential measurements from the same CdS (**c**) and CdS/WO_x_ (**d**) electrodes acquired under identical conditions; all experiments were undertaken using aqueous Na_2_SO_4_ (0.5 M) electrolyte and white light backside-illumination at a power density of 99.3 mW cm^−2^, and photocurrent testing was performed at 0 V versus Ag/AgCl (3.0 M). Where required, the annotations “On” and “Off” indicate the time or potential at which the LED source was switched. In addition to demonstrating an initial enhancement of photocurrent relative to CdS alone, the CdS/WO_x_ sample exhibited photocapacitive characteristics at the onset and termination of illumination, as evidenced by the “saw-tooth” form of the photocurrent response and the low rate at which open-circuit potential was established and removed.

Placing the sample electrodes in a three-electrode configuration with an Ag/AgCl (3.0 M) reference electrode and platinum counter electrode, the transient photocurrent response of each was tested at an applied potential of 0 V in aqueous Na_2_SO_4_ electrolyte (0.5 M) under white light illumination of radiant flux density 99.3 mW cm^−2^; the form of the LED source spectrum is provided in Fig. S3a of the Supplementary Information, alongside details regarding calculation of the radiant flux density. In the case of CdS, for which the photocurrent behaviour at 0 V versus Ag/AgCl (3.0 M) is displayed in Fig. 8a, an instantaneous anodic response was observed upon turn-on of the LED source, followed by near-instant relaxation to zero current when illumination was removed. As shown by Fig. 8b, analogous testing of the CdS/WO_x_ sample yielded an initial enhancement of the peak photocurrent with respect to CdS, while capacitive charging and discharging of the electrode occurred during cycling of the LED illumination. It is additionally apparent, however, that photocurrent from the CdS/WO_x_ sample deteriorated more rapidly than in the case of CdS, exhibiting an exponential decay over the course of the experiment.

The stability of each sample may be addressed by considering its surface reactions with species present in the electrolyte. Diminishing photocurrent has been witnessed elsewhere for CdS electrodes in aqueous Na_2_SO_4_^119,120^, and the phenomenon is associated with the aforementioned photocorrosion of CdS by photoinduced holes due to an insufficient rate of hole-scavenging by reducing species; this effect is further evidenced by transient spikes at the start and end of each photocurrent pulse, which are characteristic of surface charge accumulation^121–123^. Degradation of the CdS sample is therefore attributable to the choice of electrolyte, and in particular the absence of effective hole-scavengers: SO_4_^2−^ anions are not readily oxidised on CdS surfaces due to the high reduction potentials of the SO_4_^−^˙/SO_4_^2−^ and S_2_O_8_^2−^/SO_4_^2−^ redox couples^124,125^, and hole-removal was therefore dependent on the slow oxidation of water molecules^126^. Conversely, valence holes in WO_3_ and WO_x_ sub-oxides are typically at sufficiently high potential to instigate oxidation of SO_4_^2−^ anions;^127–129^ the diminishing photocurrent response in Fig. 8b may be instead attributed to deactivation of the WO_x_ component by peroxide species formed as a product of water oxidation at the catalyst surface^130–132^.

Of further interest in Fig. 8b is the magnitude of the photocurrent at the onset of illumination: prior to the aforementioned decay, the CdS/WO_x_ system yielded an order of magnitude increase in photocurrent relative to CdS alone. The mechanism responsible for this enhancement may be explored by addressing the corresponding photoelectrochemical behaviours of the Ta_3_N_5_, Ta_3_N_5_/WO_x_ and WO_x_ reference samples; these measurements are displayed in Fig. S4 of the Supplementary Information. While the Ta_3_N_5_ electrode generated a transient cathodic response at the onset of illumination, Ta_3_N_5_/WO_x_ correspondingly produced an almost indiscernible cathodic photocurrent and WO_x_ alone exhibited negligible photoactivity; these measurements indicate that observed differences between the CdS and CdS/WO_x_ responses cannot be ascribed to WO_x_ in isolation, and are instead indicative of an electronic interaction between the CdS and WO_x_ phases.

In addition to the enhancement of the photocurrent response, evidence of charge transfer between the components of CdS/WO_x_ is provided by the pronounced capacitive charge/discharge behaviour of the composite: following the photogeneration of electron-hole pairs in CdS, the slow recombination rate upon turn-off of the light source may be attributed to an accumulation of trapped electrons in WO_x_^133,134^. At the onset of illumination, the WO_x_ component similarly acted to retard the establishment of a stable photocurrent by extracting photoexcited electrons from CdS. Indeed, the effects of electron-trapping are further evident in open-circuit potential measurements from the CdS and CdS/WO_x_ samples, as shown in Fig. 8c,d, respectively: of the two samples, equilibrium potential was established and removed more slowly in the case of CdS/WO_x_, once again demonstrating the superior charge capacity of the composite. As in the case of photocurrent, the open-circuit potential of CdS/WO_x_ was found to diminish over the period of illumination, indicating once more that the material surface was affected detrimentally by species present in the electrolyte.

In accordance with similar systems reported elsewhere^135–137^, the transient photocurrent response of the composite may be modelled as a function of time since the onset of illumination, *t*_on_, using a bi-exponential relationship of the form

1 $$I={I}_{{\rm{f}},{\rm{o}}{\rm{n}}}(1-\exp (-\frac{{t}_{{\rm{o}}{\rm{n}}}}{{\tau }_{{\rm{f}},{\rm{o}}{\rm{n}}}}))+{I}_{{\rm{s}},{\rm{o}}{\rm{n}}}(1-\exp (-\,\frac{{t}_{{\rm{o}}{\rm{n}}}}{{\tau }_{{\rm{s}},{\rm{o}}{\rm{n}}}})),$$

where *I*_f,on_ and *I*_s,on_ respectively denote the equilibrium contributions of “fast” and “slow” processes, described by the characteristic time constants *τ*_f,on_ and *τ*_s,on_, respectively, to overall photocurrent *I*. Similarly, relaxation of the photocurrent following turn-off of the LED source may be described as a function of the time since turn-off, *t*_off_, by the formula

2 $$I={I}_{{\rm{f}},{\rm{o}}{\rm{f}}{\rm{f}}}\exp (-\frac{{t}_{{\rm{o}}{\rm{f}}{\rm{f}}}}{{\tau }_{{\rm{f}},{\rm{o}}{\rm{f}}{\rm{f}}}})+{I}_{{\rm{s}},{\rm{o}}{\rm{f}}{\rm{f}}}\exp (-\frac{{t}_{{\rm{o}}{\rm{f}}{\rm{f}}}}{{\tau }_{{\rm{s}},{\rm{o}}{\rm{f}}{\rm{f}}}})\,,$$

where processes such as the generation and recombination of electron-hole pairs in CdS and electron transfer to and from WO_x_ are once again categorised as either “fast” or “slow”, with associated time constants *τ*_f,off_ and *τ*_s,off_ and initial photocurrents *I*_f,off_ and *I*_s,off_, respectively. It is to be additionally assumed that each electrode was chemically stable in the absence of light, and therefore any time-dependence of the variables *I*_f,off_ and *I*_s,off_ in Eq. 2 is to be ignored. Conversely, *I*_f,on_ and *I*_s,on_ are to be modelled using mono-exponential relationships with respect to *t*_on_ given by

3 $${I}_{{\rm{x}},{\rm{o}}{\rm{n}}}={A}_{{\rm{x}},{\rm{o}}{\rm{n}}}({I}_{0}+{I}_{{\rm{t}}}\exp (-\frac{{t}_{{\rm{o}}{\rm{n}}}}{{\tau }_{{\rm{d}}}})),$$

where the suffix “x” must be substituted for either “f” or “s”, *A*_x,on_ is a multiplicative constant that may differ between fast and slow processes, and the constants *I*_0_, *I*_t_ and *τ*_d_ are assumed independent of process and time. Recognising that *τ*_d_ is likely to be much larger than *τ*_f,on_ and *τ*_s,on_, Eq. 1 reduces to a form proportional to Eq. 3 in the limit of large *t*_on_; moreover, one may impose the arbitrary condition that *A*_f,on_ and *A*_s,on_ sum to unity, whereupon *I*_0_, *I*_t_ and *τ*_d_ remain as the only free variables within the approximation. In this way, three of the seven free variables in Eq. 1 may be determined prior to its application as a fitting function for transient response measurements close to the onset of illumination.

**Figure 9:**
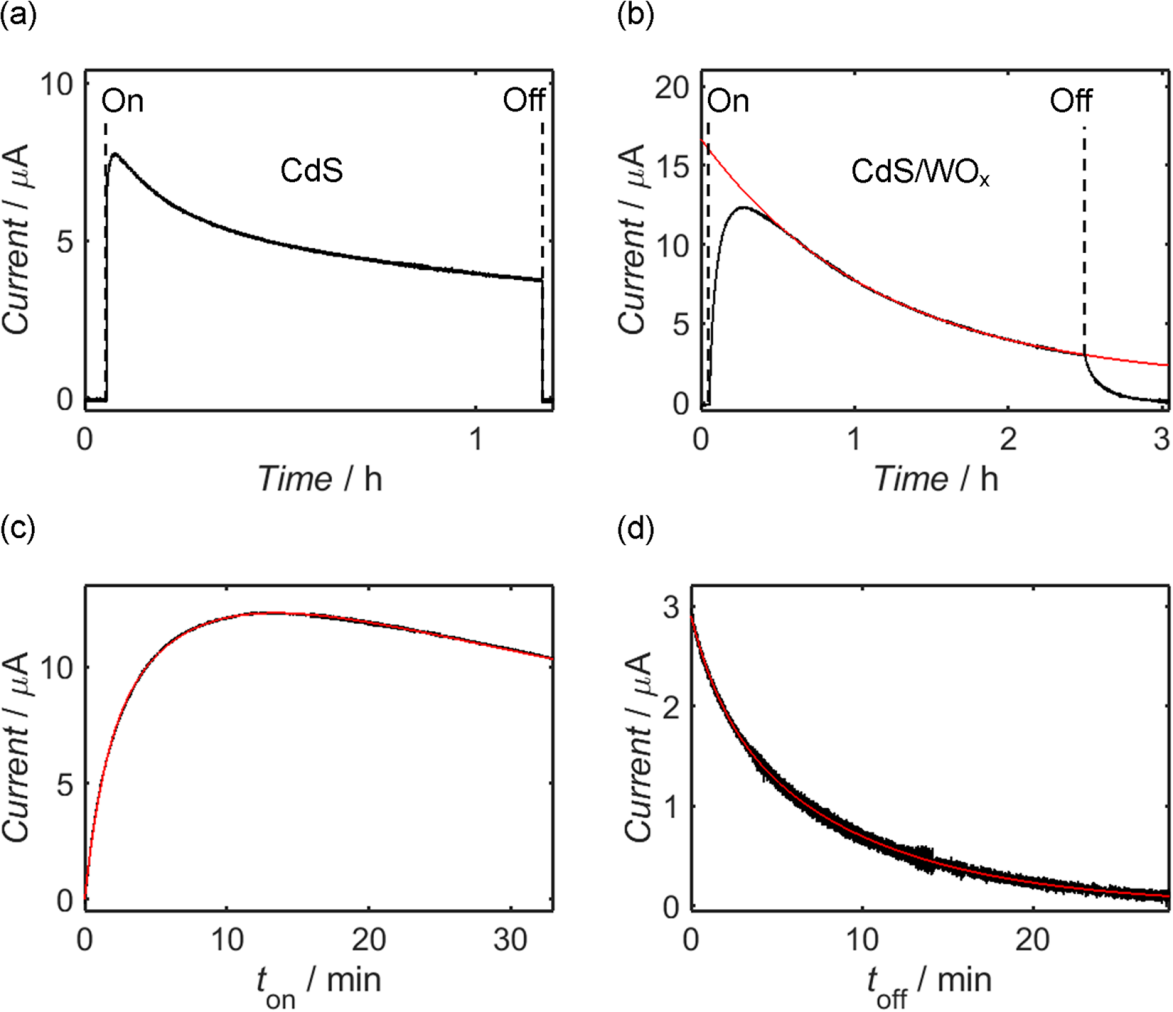
Measurement of the photocurrent responses of CdS (**a**) and CdS/WO_x_ (**b**) at 0 V versus Ag/AgCl (3.0 M) in a three-electrode configuration with a platinum mesh counter electrode and aqueous Na_2_SO_4_ (0.5 M) electrolyte, with more detailed fitting of the response (**c**) and relaxation (**d**) curves of CdS/WO_x_ shown respectively as functions of the time since turn-on, *t*_on_, and the time since turn-off, *t*_off_, of the illumination; backside-illumination was provided by a white LED source at a power density of 99.3 mW cm^−2^. In the case of the CdS/WO_x_ electrode, the decay of the photocurrent during prolonged illumination (**b**) is modelled by a mono-exponential fitting function described by Eq. 1 in the limit of large *t*_on_ (the fit has been applied over *t*_on_ values greater than 2,000 s), while the photocurrent response (**c**) and relaxation (**d**) curves of this sample are fitted according to the full bi-exponential relationships given by Eqs 1, 2, respectively; all fitting curves are plotted as solid red lines, and the annotations “On” and “Off” indicate the switching times of the LED source.

The decay of photocurrent during prolonged illumination of the CdS and CdS/WO_x_ samples is shown in Fig. 9a,b, respectively, with the form of the diminishing response fitted in the latter case using Eqs 1, 3 for *t*_on_ values greater than 2,000 s; from this fit, a *τ*_d_ estimate of 4167.3 ± 1.5 s is acquired for the CdS/WO_x_ electrode, alongside *I*_0_ and *I*_t_ values of 1.2218 ± (1.7 × 10^−3^) μA and 14.7356 ± (1.0 × 10^−3^) μA, respectively. The transient response of the CdS/WO_x_ system has been subsequently modelled using the exact form of Eq. 1, as shown in Fig. 9c; in conjunction with the three estimated variables from Fig. 9b, the depicted curve is well-characterised by respective *τ*_f,on_ and *τ*_s,on_ values of 81.24 ± 0.12 s and 381.5 ± 0.2 s, with *A*_f,on_ and *A*_s,on_ estimated as 0.3637 ± (5 × 10^−4^) and 0.6363 ± (5 × 10^−4^), respectively. Similarly, the relaxation curve in Fig. 9d has been fitted by Eq. 2 using values of 110.9 ± 1.0 s and 547.2 ± 1.0 s for *τ*_f,off_ and *τ*_s,off_, respectively, in addition to respective values of 0.827 ± (5 × 10^−3^) μA and 2.077 ± (5 × 10^−3^) μA for *I*_f,off_ and *I*_s,off_.

Having explored the photocapacitive behaviour of the CdS/WO_x_ composite, it is important to compare the characteristics to corresponding measurements from similar materials. Unfortunately, precise comparison with previous CdS/WO_x_ composites is difficult because, to the authors’ knowledge, researchers have yet to explore the photocapacitive properties of this material combination; nevertheless, there exist studies which briefly report the photocurrent response of CdS/WO_x_ composites, albeit without any focus on the self-charging behaviour^55,57,105^. Relative to these previous examples, the present composite exhibited a superior initial photocurrent response and a similar response after multiple cycles: under similar experimental conditions, other CdS/WO_x_ composites typically yielded photocurrents of around 10 µA cm^−2^. Additionally, the rate of discharge in the cited works was significantly more rapid than in the present case: as shown by Fig. 9d, a current was measured from the CdS/WO_x_ composite more than twenty minutes after final illumination. In the work by Zhang *et al*.^57^, by contrast, near-total discharge was achieved within a period of approximately 20 s, whereas the material synthesised by Jin *et al*.^55^ exhibited a discharge time of approximately 60 s. Other binary composites produced similar short-circuit discharge currents, with discharge times of less than 60 s typical for these materials^27–29,138–140^. In the majority of the cited examples, including those corresponding to CdS/WO_x_ composites, the discharge current decreased to less than half of its original value almost instantly upon turn-off of the light source, in stark contrast to the comparatively gradual rate of discharge shown in Fig. 9d.

When investigating the behaviour of capacitive systems, a conventional approach is to estimate the specific capacitance of an electrode through cyclic voltammetry or galvanostatic charge/discharge measurements^141–145^. Though useful, it has been argued elsewhere that these experiments are unfortunately misleading when a system behaves as a current source in addition to its supercapacitive properties^146^. Moreover, due to the rudimentary drop-casting approach adopted for electrode preparation in the present study, sample inhomogeneity and incomplete adhesion of the sample materials to the underlying FTO-coated glass prohibited meaningful estimation of the specific capacitance. It is for these reasons that the present investigation is focussed on the form of the photoresponse under short-circuit and open-circuit conditions, in addition to addressing the electronic mechanisms responsible for the observed behaviours.

## Conclusions

By examining the photocurrent response and open circuit potential of a CdS/WO_x_ composite under white LED illumination, the photocapacitive properties of the material have been demonstrated. At a power density of 99.3 mW cm^−2^, the sample out-performed existing CdS/WO_x_ composites in photoelectrochemical tests, exhibiting a similar photocurrent response after multiple illumination cycles but a markedly superior ability to store photoinduced charge: short-circuit current persisted for more than twenty minutes after final illumination, compared to less than a minute in previous examples from the literature.

Based on the electronic band edge positions deduced from XPS, UPS and UV-Vis diffuse reflectance measurements, it has been surmised that the measured charge-accumulation in CdS/WO_x_ resulted from the trapping of photoexcited electrons from CdS following their transfer to the conduction band of WO_x_. Moreover, the enhancement of photocurrent with respect to a CdS control sample may be attributed in part to the quenching of valence holes at the CdS/WO_x_ interface, thereby extending the mean lifetime of photoexcited electrons by suppressing electron-hole recombination within the CdS component. These deductions are supported by similar measurements from a Ta_3_N_5_/WO_x_ reference sample, which was synthesised using an analogous solvothermal protocol: the Ta_3_N_5_/WO_x_ composite and separate Ta_3_N_5_ and WO_x_ reference electrodes produced no significant photocurrent response under LED illumination, indicating that the photocapacitive characteristics of CdS/WO_x_ resulted from a synergistic electronic relationship between its constituents and not from one component in isolation.

With solar technologies likely to play a critical role in future energy production, self-charging materials are a potentially invaluable commodity. In showcasing the photocapacitive behaviour of a CdS/WO_x_ composite, the present study provides a seminal insight into the promise of this material combination. Building on this work, one might conceive of a layered, self-charging solar cell that utilises CdS for the photogeneration of electron-hole pairs and an adjoining WO_x_ layer for subsequent charge storage. Furthermore, since the self-charging performance of the present composite was superior to other CdS/WO_x_ composites reported in the literature, it is likely that further improvement might be achieved through optimisation of the synthesis protocol; it would be instructive, for instance, to explore the effects of varying the relative quantities of the two constituents or, alternatively, the size or shape of the CdS or WO_x_ particles.

Within this seminal investigation into CdS/WO_x_ composites for self-charging solar applications, the viability of the system has been demonstrated in a predominantly qualitative manner. There remains significant scope for development with respect to the uniformity of the working electrode and its mechanical stability. Mitigation of these problems and more quantitative measurement of properties such as the specific capacitance, as well as examination of the factors affecting the chemical stability of the composite, are left to future work; nevertheless, the present study serves as a valuable foundation for additional research.

## Methods

### CdS nanoparticles

CdS nanoparticles were grown using a solvothermal technique adapted from the literature^147^. Cadmium(II) acetate dihydrate (10 mmol) and thiourea (20 mmol) were dissolved in deionised water (60 ml), before the solution was transferred to a PTFE cup of 125 ml capacity which was in turn secured inside a stainless steel Parr acid digestion bomb. The vessel was heated in a muffle furnace to 180 °C at a rate of 10 °C min^−1^, and was maintained at this temperature for ten hours. After cooling naturally to room temperature, the resulting orange precipitate was cleaned through repeated centrifugation into deionised water and then absolute ethanol, before being dried overnight at 80 °C and subsequently ground into a fine powder using a pestle and mortar.

### CdS/WO_x_ composite

Synthesis of the CdS/WO_x_ composite was achieved using a second solvothermal step. CdS nanoparticles (20 mM) were added to a solution of tungsten(VI) chloride (20 mM) in 4:1 v/v mixture of absolute ethanol and ethylene glycol. The resulting suspension was transferred to a PTFE cup of 125 ml capacity and secured inside a stainless steel Parr acid digestion bomb. Using a muffle furnace, the suspension was heated at a rate of 10 °C min^−1^ to 180 °C, and this temperature was held constant for a period of 24 hours. The vessel was subsequently cooled naturally to room temperature, and the dark-green precipitate was obtained by centrifuging repeatedly into deionised water and then absolute ethanol, followed by drying overnight at 80 °C. The dried material was finally ground into a fine powder using a pestle and mortar.

### Ta_3_N_5_, WO_x_ and Ta_3_N_5_/WO_x_ reference samples

To serve as reference samples, nanoparticles of tantalum(V) nitride, Ta_3_N_5_, tungsten(VI) sub-oxide, WO_x_, and a nanocomposite of tantalum(V) nitride and tungsten(VI) sub-oxide, Ta_3_N_5_/WO_x_, were synthesised using procedures described elsewhere^59^. In brief, the Ta_3_N_5_ nanoparticles were prepared inside a tube furnace by annealing a mixture of tantalum(V) chloride (2.0 mmol), sodium chloride (2.8 mmol) and potassium chloride (1.2 mmol) at 800 °C for ten hours under continuous flow of ammonia gas. The Ta_3_N_5_/WO_x_ composite was subsequently obtained using a technique analogous to that described for CdS/WO_x_, with the CdS substituted for Ta_3_N_5_ (3.8 mM) and tungsten(VI) chloride present at a slightly increased concentration of 22.8 mM. All other reaction conditions, including the heating parameters, the composition and volume of the solvent, and the centrifugation steps used to clean the final precipitate, remained the same as in the CdS/WO_x_ case. To prepare the WO_x_ reference sample, an ethanolic solution of WCl_6_ (12.6 mM, 70 ml) was transferred to a 125 ml PTFE cup and heated at a rate of 10 °C min^−1^ in the 125 ml stainless steel Parr acid digestion bomb to a temperature of 180 °C, which was maintained for 24 hours. The resulting flocculent blue precipitate was centrifuged into ethanol and then deionised water, before drying overnight at a temperature of 80 °C. All reference materials were ground into a fine powder using a pestle and mortar.

### SEM and EDX characterization

For SEM analysis, powders of CdS and CdS/WO_x_ were deposited onto separate adhesive carbon tabs and examined using a Hitachi S4800 FE-SEM, with the accelerating voltage set to 10 kV and an emission current of 10 µA. Within the analysis chamber of the FE-SEM instrument, the elemental composition of each sample was measured at 5,000-times magnification using an Oxford Instruments Silicon Drift X-Max energy-dispersive X-ray (EDX) detector with 50 mm^2^ active area; the spectrum was recorded at an accelerating voltage of 20 kV and an emission current of 10 µA, and was subsequently analysed using Oxford Instruments INCA EDX software.

### Phase identification

The crystal structures of CdS and CdS/WO_x_ were investigated through X-ray diffractometry carried out using a Bruker D8 Advance X-ray diffractometer. For these measurements, a small quantity of each material was transferred into a special glass capillary of 0.5 mm diameter, and the diffractograms were acquired using Cu-Kα radiation in conjunction with a Ni filter. The step-size employed was 0.015°, with an acquisition time of 10 s per step and a 2*θ* range of 10° to 90°.

### Band-gap quantification

UV-Vis diffuse reflectance measurements were carried out for samples of CdS and CdS/WO_x_ by transferring each powder to a spring-loaded powder cell in an Agilent Cary 100 UV-Vis spectrophotometer, with the spectra referenced to a Labsphere Spectralon diffuse reflectance standard. The spectra were recorded using a quartz-iodide lamp over a wavelength range of 400–800 nm, with a step size of 1 nm and 100 ms per step. From the diffuse reflectance spectra, Tauc plots were constructed using a Matlab program, which also provided an estimate of the band-gap by extrapolating a line-of-best-fit through the point of steepest gradient on the absorption cut-off curve.

### XPS and UPS measurements

The surface chemical composition of each sample was investigated through XPS analysis. Spectra were recorded using a Kratos Axis Supra system employing a monochromated Al-Kα X-ray source; for these measurements, the CdS and CdS/WO_x_ powders were each compressed using a two-tonne press into a circular pellet of 5 mm diameter and mounted on conductive copper tape. To maximise electrical contact between the sample plate and the pellet surface, copper clips were screwed in place over a portion of each pellet, leaving the remainder exposed for measurement. Survey spectra were recorded across binding energies of 0–1200 eV using pass energy 160 eV, dwell time 100 ms and step size 1 eV. With the exceptions of the Cd 3d peak of CdS and the W 4f, O 1 s and Cd 3d peaks of CdS/WO_x_, core level peaks of interest were measured using pass energy 20 eV, dwell time 1500 ms and step size 50 meV, and averaging over three sweeps; in the case of the other core level peaks of interest, the same pass energy, step size and number of repeat scans were used, but the dwell time was reduced to a value of 500 ms for the Cd 3d peak of CdS and a dwell time of 1000 ms was employed for the W 4f, O 1 s and Cd 3d peaks of CdS/WO_x_. All XPS measurements were performed over a rectangular area of 700 × 300 µm.

Surface charge compensation during the XPS measurements was achieved using a charge neutraliser operating at a charge balance potential of 3.3 V, filament bias 1.0 V and filament current 0.4 A. The spectra were “carbon-corrected”, with the binding energy scale shifted by the value required to reference the C 1 s peak to 284.8 eV, the accepted value for the predominant sp^3^ carbon environments of adventitious organic contaminants^89^. Each core level peak was deconvoluted using Gaussian-Lorentzian product functions, while the contribution of the secondary electron background was modelled using a Shirley-type function. In the case of a doublet, the relative areas of the two components were constrained following consideration of spin population statistics, and the requisite spin-orbit splitting was imposed accordingly. With the exception of the W 4f signal of CdS/WO_x_, the full widths at half maximum of components within a particular peak were constrained to be equal; conversely, it was ensured that 4f_5/2_ and 4f_7/2_ components associated with a particular chemical environment of the W 4f peak were set equal, but components corresponding to different environments were allowed different peak widths. Moreover, while most of the peak fitting was achieved using Gaussian-Lorentzian product functions with 30% Lorentzian character, those of the W 4f signal possessed 80% Lorentzian character due to the greater asymmetry required for the fit. Atomic ratios of different elements were calculated by dividing the areas of the fitted components by the relevant relative sensitivity factors.

To determine the valence band maximum position of each sample, XPS measurements were performed at binding energies close to the Fermi edge position at 0 eV. These scans were undertaken using pass energy 20 eV, dwell time 1500 ms and step size 50 meV, averaging over three sweeps. The ionisation potential of each sample was estimated through use of UPS; to prepare the pelletised samples for these measurements, surface contamination was first removed by bombarding a 4 × 4 mm area with 1000+ atom argon ion clusters of total energy 10 keV for a period of 120 s. Measurements were subsequently carried out using a He I source (21.22 eV photon energy) over a kinetic energy range of 0.4–23.0 eV, using pass energy 5 eV, step size 25 meV, 60 ms dwell time and a 55 µm diameter aperture, followed by an additional scan in each case between kinetic energies of 8.0 eV and 23.0 eV, with the same pass energy, step size and dwell time but an aperture of diameter 110 µm. All UPS measurements employed a −9 V sample bias potential.

### Photoelectrochemical testing

For measurements of the electrical response to white light illumination under both short-circuit and open-circuit conditions, electrodes were prepared using fluorine-doped tin oxide (FTO)-coated glass with a sheet resistance of 7 Ω per square. Prior to fabrication, the FTO-coated glass was cut into a 50 × 25 mm rectangle and cleaned thoroughly by ultra-sonication in acetone followed by isopropanol. After masking a 15 × 15 mm square area with scotch tape and providing electrical contact to the top of the electrode via a copper wire secured with copper tape, the remainder of the electrode was electrically isolated using a UV-cured glass-fibre epoxy resin. The scotch tape was subsequently removed and the exposed area cleaned once more with acetone and isopropanol. To deposit the sample material, sample powder (10 mg) was first suspended in absolute ethanol (1 ml), with ultra-sonication employed to ensure a homogenous suspension. Maintaining the electrode at a temperature of 70 °C on a hot-plate, the material was sequentially layered onto the exposed area through stepwise drop-casting of the suspension, allowing the material to dry completely between successive steps.

White light illumination was provided by a custom-built light box containing a 40 W LED source, which provided a radiant flux density of 99.3 ± 0.6 mW cm^−2^ to the sample. Measurement of the luminous flux density was carried out using an Extech EA33 EasyView light meter, while the form of the spectrum was recorded by an Ocean Optics USB 2000+ spectrometer. Estimation of the radiant flux density from these measurements was achieved through use of the CIE standard photopic luminous efficacy function^148–151^; further details regarding this calculation and the measured form of the LED spectrum are provided by Fig. S3 of the Supplementary Information and the accompanying discussion.

For all photoelectrochemical measurements, electrodes were immersed in an aqueous solution of Na_2_SO_4_ (0.5 M) and the tests were performed using a three-electrode setup with an Ag/AgCl (3.0 M) reference and platinum mesh counter electrode. In each case, the FTO-coated glass working electrode was configured for back-side illumination. Both the reference and counter electrodes were rinsed thoroughly with deionised water between testing of different sample electrodes, and the aqueous Na_2_SO_4_ electrolyte was also replaced.

To investigate the transient photocurrent response of the samples, a potential difference of 0 V was applied between the working and reference electrodes and the current passing between the working and counter electrodes was measured during white light illumination. Initial measurements were carried out using chopped LED illumination with a period of approximately 330 s, before the sample electrode was rinsed in deionised water, allowed to dry naturally and left overnight in darkness. The electrode was then reintroduced to the three-electrode setup as before with a fresh batch of electrolyte solution, and exposed to the LED light continuously for several hours to observe the photocurrent variation during prolonged illumination. Photocurrent response and relaxation curves were fitted using a non-linear regression protocol carried out by a Matlab program, with the standard error in each fitting parameter calculated from the square root of the diagonalised covariance matrix generated by the regression function.

After returning the setup to darkness and allowing the photocurrent to diminish to a stable value close to zero, the open-circuit potential of each sample electrode was investigated. With the current between the sample and counter electrodes constrained to zero, the LED remained off until a stable potential difference was attained between the sample and reference electrodes. Once stabilised, the system was illuminated continuously for several hours to observe the variation of the potential difference as a function of exposure time, and the relaxation of the system was subsequently explored after turn-off of the LED source.

## Supplementary information

Supplementary Information
