# Supplementary material for: Photocapacitive CdS/WOx nanostructures for solar energy storage
Source: Sci Rep. 2019 Aug 9;9:11573. doi: 10.1038/s41598-019-48069-5 (PMC6688992; doi:10.1038/s41598-019-48069-5)
Supplement: Supplementary file 1 — Supplementary Information [file 41598_2019_48069_MOESM1_ESM.pdf]

Electronic Supplementary Information

For

Photocapacitive CdS/WO_x nanostructures for solar energy storage

Daniel R. Jones[1], Robert Phillips[1], William J. F. Gannon[1], Bertrand Rome[1], Michael E. A. Warwick[1] and Charles W. Dunnill*[1]

[1]Energy Safety Research Institute, Swansea University Bay Campus, Swansea, SA18EN, UK.

*Correspondence to c.dunnill@swansea.ac.uk

Analysis of bulk elemental composition

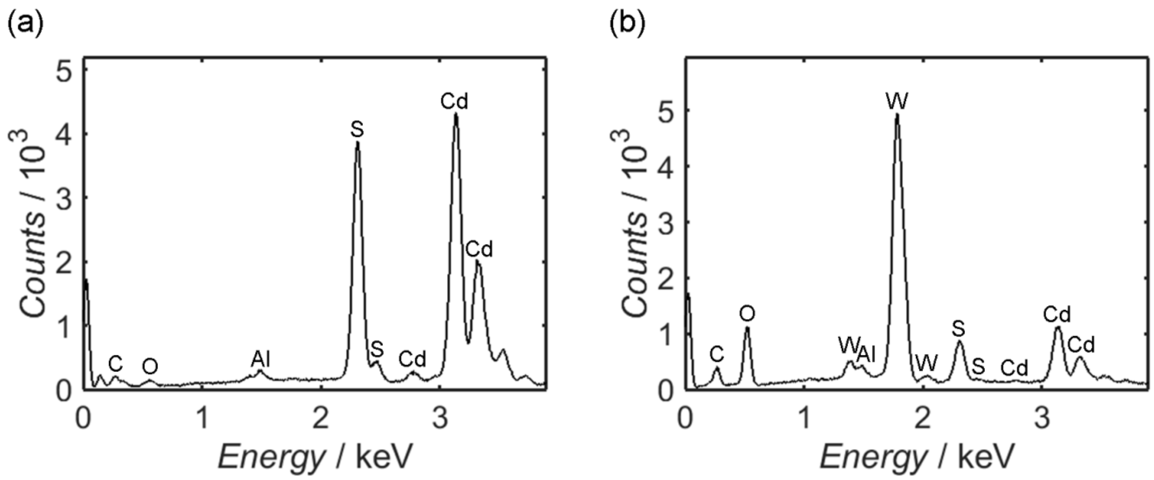

Fig. S1. EDX measurements from the CdS (**a**) and CdS/WO_x (**b**) samples (deposited on adhesive carbon tabs), recorded at an accelerating voltage of 20 kV and emission current 10 μA; both spectra suggest a Cd/S atomic ratio of approximately one, while the measurements from CdS/WO_x additionally verify the presence of a WO_x phase and organic contaminants. The source of the Al content in each spectrum is unclear, and the element was not identified in XPS measurements from either sample.

	**Carbon tab**	**CdS**	**CdS/WO_x**
**Element**	**Atomic percentage**		
**C**	92.41	35.85	24.64
**O**	6.97	2.99	55.42
**S**	0.21	29.28	4.87
**Cd**	0.00	30.53	5.16
**W**	0.00	0.00	9.10
**Al**	0.06	1.34	0.81
**Na**	0.32	0.00	0.00
**Si**	0.03	0.00	0.00

Table S1. Atomic percentage measurements obtained from EDX spectra of the CdS and CdS/WO_x samples, with the measured elemental composition of the underlying adhesive carbon tab provided for reference.

Fitting of UPS spectra

The UPS measurements shown in Fig. 5**c** and **d** of the paper are instrumental in determining the band structure of CdS/WO_x. More specifically, estimation of the secondary electron onset, $E_{k,SEO}$, and valence band maximum, $E_{k,VB}$, of both CdS and the composite allows calculation of the ionisation energy in each case, defined as the energy of the valence band edge relative to the vacuum level, E_{vac}.

To estimate $E_{k,SEO}$, a Matlab program is used to plot a linear fit through the points of steepest gradient on the onset curve, while a second line is similarly extrapolated through the data defining the baseline at lower values of kinetic energy; these fits are depicted as dashed lines in Fig. S2**a** and **b** for CdS and CdS/WO_x, respectively. In both cases, $E_{k,SEO}$ is taken as the kinetic energy at the point of intersection between the two linear fits.

The energy of the valence band maximum is estimated in a similar fashion. Illustrated as dashed lines in Fig. S2**c** and **d**, which correspond to CdS and CdS/WO_x, respectively, are linear fits through the points of steepest gradient close to the valence band edge and through the baseline at higher values of kinetic energy; as in the case of $E_{k,SEO}$, the kinetic energy at the point of intersection between the two lines is assumed equal to $E_{k,VB}$.

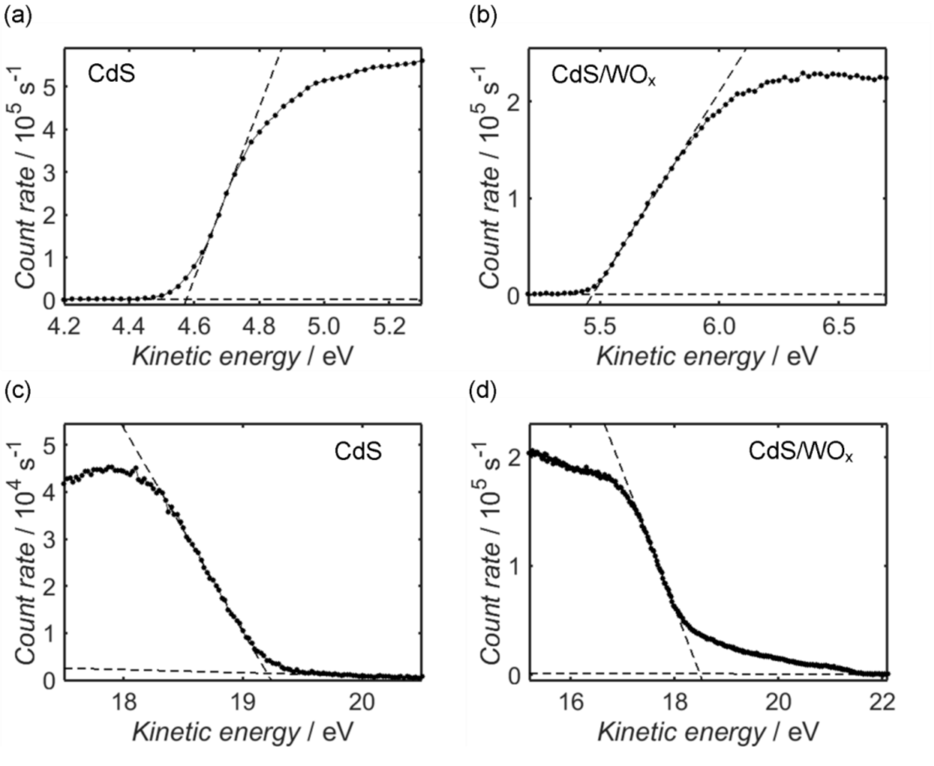

Fig. S2. Secondary electron onsets in the UPS spectra of CdS (**a**) and CdS/WO_x (**b**), in addition to the UPS measurements close to the valence band maximum of CdS (**c**) and CdS/WO_x (**d**). Linear fits to the onset and valence band edge features have been constructed through the points of maximum gradient, as determined by a Matlab program, and are plotted as dashed lines. Also shown as dashed lines are linear fits through the baselines of each measurement set; the kinetic energy at the point of intersection determines the value of either the secondary electron onset, $E_{k,SEO}$, or the valence band maximum, $E_{k,VB}$.

Estimation of incident light intensity

To calculate the radiant flux density, Φ_e, incident on a system from knowledge of the luminous flux density, Φ_v, one must address the relationships between these two quantities and the measured light spectrum, $\Phi_{e,\lambda}$, where the suffix denotes a dependence on the photonic wavelength, λ. The form of the light spectrum may be measured at an arbitrary position relative to the light source, provided that there is no spectral variation between the selected position and the location of the experimental system. In the present case, saturation of the spectrometer precluded measurement of $\Phi_{e,\lambda}$ at the system location, so the measurement was instead carried out at a greater distance from the LED source; the resulting spectrum is displayed as a function of wavelength in Fig. S3**a**.

In addition to determining the form of $\Phi_{e,\lambda}$, the value of Φ_v was also measured at the system location; these variables are related via the equation

$$\Phi_v = C \int_0^{\infty} \eta_\lambda \Phi_{e,\lambda} \, d\lambda, \qquad \text{(S1)}$$

where C is a constant and η_λ is the standard photopic luminous efficacy function, plotted in Fig. S3**b**, which characterises the sensitivity of the human eye and provides the basis for the definition of the lumen unit. Having already measured both Φ_v and $\Phi_{e,\lambda}$, the constant C may be estimated directly from (S1). Finally, Φ_e is given by

$$\Phi_e = C \int_0^{\infty} \Phi_{e,\lambda} \, d\lambda, \qquad \text{(S2)}$$

and may therefore be calculated from the estimate of C and the measured form of $\Phi_{e,\lambda}$. For the photoelectrochemical experiments in the present study, the measured Φ_v value of 325,250 ± 2000 lux yielded a Φ_e estimate of 99.3 ± 0.6 mW cm^{-2}, equivalent to an intensity of 1.0 Suns.

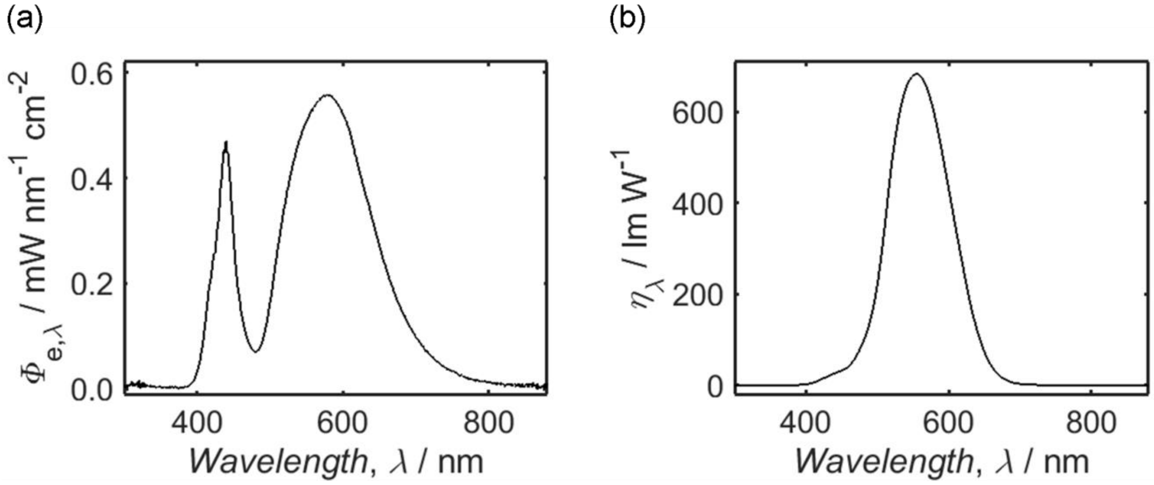

Fig. S3. Spectrum of the LED source measured using an Ocean Optics USB 2000+ spectrometer at an arbitrary position relative to the sample location (**a**), and the CIE standard photopic luminous efficacy function, η_λ (**b**).

Photocurrent responses of reference samples

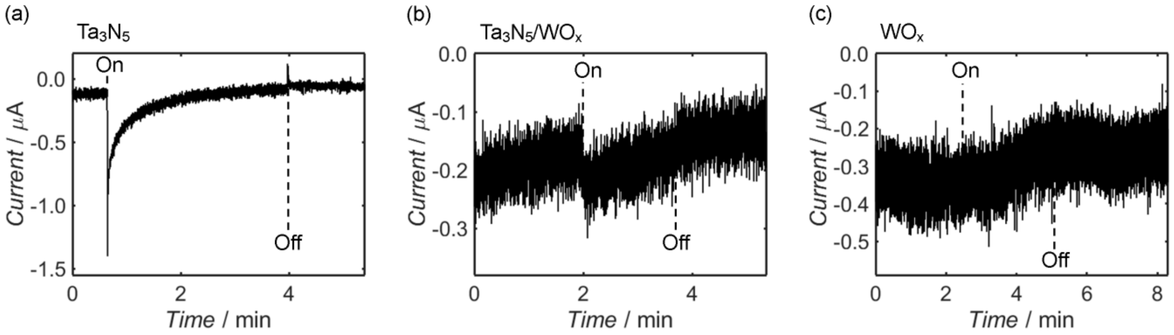

Fig. S4. Transient photocurrent response measurements from Ta_3N_5 (**a**), Ta_3N_5/WO_x (**b**) and WO_x (**c**) on FTO-coated glass in a three-electrode configuration with a platinum mesh counter-electrode and Ag/AgCl (3.0 M) reference; aqueous Na_2SO_4 (0.5 M) was used as the electrolyte and a potential of 0 V was applied to the sample with respect to the reference electrode. Each sample was backside-illuminated by an LED source of power density 99.3 mW cm^{-2}. The Ta_3N_5 sample exhibited a diminishing cathodic current upon turn-on of the LED source, while a near-negligible cathodic photocurrent was measured in the case of Ta_3N_5/WO_x. When used alone, WO_x demonstrated negligible photoactivity in the present experimental setup.
